# Transactions of the New York Academy of Medicine

**Published:** 1892-06

**Authors:** 


*Transactions of the New York Academy of Medicine.* Second Series. Vol. VII. Pp. 461. Printed for the Academy. 1891.

This volume, which deals with subjects that are almost wholly medical, maintains the high level of excellence which we are accustomed to associate with the New York Academy, and most of the contributions will repay careful study. There are two papers on Abdominal Surgery, and one on the Surgery of the Spinal Cord. We may perhaps, without being invidious, point out the papers which seem to us especially valuable. There is a description of a new apparatus, by Dr. Seibert, for artificial feeding of infants, in which the milk is sterilised in the bottles; the apparatus is of great simplicity, and so cheap as to be within reach of all. There are papers on Influenza and its most fatal complication, Pneumonia, and an important contribution to the study of Infantile Cerebral Palsy, based on analysis of 140 cases, by Sachs and Peterson. The subject most fully worked out in this book is that of Reflex Neuroses, or peripheral irritation as a cause of nervous affections. The eye, throat, nose, pelvic organs, and teeth are all considered from this point of view. From the omission of the ear, we should conclude that it is not a frequent source of reflex irritation. We may quote Prof. Allen Starr's caution, in his article on Reflex Neuroses in general, that in cases of disease put down to peripheral irritation, the disease may be in the morbid condition of the nervous centre, which is too readily affected by changes at the periphery.

Among the rest are four papers on Hydrophobia and Pasteur's Prophylaxis for Rabies, and two on Optic Neuritis in connection with orbital and intracranial disease respectively.

